# Differentially Expressed microRNAs and Target Genes Associated with Plastic Internode Elongation in *Alternanthera philoxeroides* in Contrasting Hydrological Habitats

**DOI:** 10.3389/fpls.2017.02078

**Published:** 2017-12-05

**Authors:** Gengyun Li, Ying Deng, Yupeng Geng, Chengchuan Zhou, Yuguo Wang, Wenju Zhang, Zhiping Song, Lexuan Gao, Ji Yang

**Affiliations:** ^1^Key Laboratory for Biodiversity Science and Ecological Engineering, Ministry of Education, Fudan University, Shanghai, China; ^2^Shanghai Key Laboratory of Plant Functional Genomics and Resources, Shanghai Chenshan Botanical Garden, Shanghai, China; ^3^Institute of Ecology and Geobotany, Yunnan University, Kunming, China

**Keywords:** microRNA, dynamic expression, time course analysis, phenotypic plasticity, *Alternanthera philoxeroides*

## Abstract

Phenotypic plasticity is crucial for plants to survive in changing environments. Discovering microRNAs, identifying their targets and further inferring microRNA functions in mediating plastic developmental responses to environmental changes have been a critical strategy for understanding the underlying molecular mechanisms of phenotypic plasticity. In this study, the dynamic expression patterns of microRNAs under contrasting hydrological habitats in the amphibious species *Alternanthera philoxeroides* were identified by time course expression profiling using high-throughput sequencing technology. A total of 128 known and 18 novel microRNAs were found to be differentially expressed under contrasting hydrological habitats. The microRNA:mRNA pairs potentially associated with plastic internode elongation were identified by integrative analysis of microRNA and mRNA expression profiles, and were validated by qRT-PCR and 5′ RLM-RACE. The results showed that both the universal microRNAs conserved across different plants and the unique microRNAs novelly identified in *A. philoxeroides* were involved in the responses to varied water regimes. The results also showed that most of the differentially expressed microRNAs were transiently up-/down-regulated at certain time points during the treatments. The fine-scale temporal changes in microRNA expression highlighted the importance of time-series sampling in identifying stress-responsive microRNAs and analyzing their role in stress response/tolerance.

## Introduction

The ability of organisms to produce distinct phenotypes under different environmental conditions is referred to as phenotypic plasticity (Schlichting, 1986; Sultan, 2000; Pigliucci, 2005), which is crucial for organisms to survive in a world that is constantly changing, especially when the environment changes quickly (Ghalambor et al., 2007). It is usually considered that plants have greater plasticity than animals for their sessile lifestyle (Bradshaw, 1965; Sultan, 2000; Borges, 2008). Plants are forced to cope with local environmental fluctuations by plastic modifications in morphology, physiology, behavior or life history traits, to overcome the challenges from changing environments and increase their niche breadth (Richards et al., 2006; Ghalambor et al., 2007). Over the past two decades, the molecular and cellular mechanisms of environmental perception and gene regulation underlying various plastic responses in plants have been an explicit focus of the ecological developmental (Eco-Devo) research, with the primary goal of characterizing the developmental genetic pathways supporting the ability to respond plastically to environment changes (Sultan, 2007, 2010; Gilbert, 2016). Understanding the regulatory events informed by both external and internal environmental signals and identifying genetic elements implicated in environmentally contingent development will provide key insights to the immediate tolerance and potential evolutionary resilience of organisms to environmental fluctuations (Sultan, 2007, 2010; Gilbert, 2016).

Investigating the development of plastic traits in ecological context has revealed an association between environmentally contingent phenotypic expression and the environmental sensitivity of gene expression (Morris and Rogers, 2014). This environmental sensitivity of gene expression is, in turn, driven by the flexibility of epigenetic gene regulation in fluctuating environments (Bossdorf et al., 2008; Richards et al., 2017). Epigenetic variations triggered by environmental perturbations can lead to inducible phenotypic changes by altering transcriptional profiles and eventually altering ontogenetic trajectory in response to environmental variation, even in the complete absence of genetic variability (Kalisz and Kramer, 2008; Aubin-Horth and Renn, 2009), and thus serve as a mechanistic link between genes and environment (Schlichting and Smith, 2002; Morris and Rogers, 2014). A suite of epigenetic mechanisms, including DNA methylation, histone modifications and small RNAs, have been shown to be able to control the temporal, spatial and abundance patterns of gene expression and RNA-translation under varied environments (Schlichting and Smith, 2002; Chinnusamy and Zhu, 2009). These epigenetic mechanisms may act separately or concomitantly (Bilichak et al., 2012; Richards et al., 2017).

microRNAs, a type of endogenous small-non-coding RNAs, have emerged as key post-transcriptional regulators of gene expression through base pairing with their complementary mRNA targets (Jones-Rhoades et al., 2006). Functional genomic studies have shown the involvement of plant microRNAs in a broad range of developmental processes (Eldem et al., 2013; Jin et al., 2013; Li and Zhang, 2016). miR165/166 have been proved to participate in SAM development, acting as mobile signal molecules for stem cell maintenance (Zhang and Zhang, 2012). miR319 mediates the change of plant leaf shape via targeting TCP transcription factors (Palatnik et al., 2003), while miR160 and miR393 play key roles in root architecture regulation via post-transcriptional modification of the auxin signal pathway genes (Wang et al., 2005; Bian et al., 2012). Both miR156 and miR172 are involved in regulation of flowering time by age-dependent pathway (Wang, 2014; Li and Zhang, 2016). Multiple microRNAs, including miR169, miR164, miR319, miR159, and miR167 function to control floral organ identity fate during flower development by regulating the spatial boundaries of expression of target genes, and to specify particular cell types during later stages of flower development (Nag and Jack, 2010). It has also been revealed that a single miRNA may have diverse regulatory roles. For example, miR159 is involved in flowering time control, leaf development, seed size and shape determination, respectively (Blazquez et al., 1998; Palatnik et al., 2003; Achard et al., 2004).

In addition to control various developmental processes, plant microRNAs also act as node molecules to coordinate the balance between plant development and environmental clues, playing critical roles in response to biotic and abiotic stresses (Sunkar et al., 2012; Zhang, 2015; Li and Zhang, 2016; Noman et al., 2017), microRNAs involved in responses to water-logging/flooding have been demonstrated in different plants. For instance, Zhang et al. (2008) and Zhai et al. (2013) identified microRNAs responding to waterlogging in maize roots. Khan et al. (2014) predicted the associated microRNAs under waterlogged conditions in sugarcane. Ren et al. (2012) found significant changes in the expression of seven conserved microRNA families and five novel microRNAs in response to flooding stress in *Populus tomentosa*. Jin et al. (2017) revealed the microRNA-mediated regulatory networks in lotus by identifying submergence-responsive microRNAs and their targets. Flooding exposes plants to severe hypoxia. Thus, microRNAs mediating hypoxic stress responses in plants were also thought to facilitate the survival and adaptation to the flooding condition by modifying gene expression, metabolic networks, and developmental processes (Moldovan et al., 2009; Licausi et al., 2011).

*Alternanthera philoxeroides* is an exotic amphibious weed that is native to South America but has now invaded into the temperate and tropical regions across the world (Julien et al., 1995; Pan et al., 2007). *A. philoxeroides* rarely produces viable seeds in its introduced range, and propagates mainly via vegetative regeneration (Julien and Stanley, 1999; Geng et al., 2007; Pan et al., 2007). Plants produced by vegetative propagation are genetically identical. However, these plants can grow in diverse habitats, from purely aquatic environments (ponds, rivers) to dry lands (Pan et al., 2007). Individuals growing in aquatic and terrestrial habitats exhibited notable morphological differences, with the plants growing in aquatic environments showing significantly longer internode length and greater stem diameter (Geng et al., 2007; Gao et al., 2010). Plasticity in internode elongation, aerenchyma formation, and other morphological and physiological traits allows the amphibious plants of *A. philoxeroides* to withstand alternative wetting and drying conditions and to proliferate in both aquatic and terrestrial environments (Geng et al., 2006, 2007; Pan et al., 2007).

As the most conspicuous responses of plants subject to submergence, enhanced internode/petiole elongation and secondary aerenchyma formation have been found in many species (Jackson and Armstrong, 1999; Van Veen et al., 2013; Yamauchi et al., 2013; Voesenek and Bailey-Serres, 2015). The molecular and cellular mechanisms underlying these responses, such as the external and internal environmental cues, signaling pathways, and genetic elements implicated in developmental outcomes, have been explored in deepwater rice (Nishiuchi et al., 2012), *Rumex* (Peeters et al., 2002), and other species (Bailey-Serres and Voesenek, 2010; Voesenek et al., 2014; Loreti et al., 2016). However, the epigenetic mechanisms underpinning submergence-induced plastic development, including the potential roles of microRNA-mediated regulation of gene expression, remain elusive. Since the transition from aquatic to terrestrial environments, or vice versa, requires timely and flexible adjustments in morphological traits, physiological processes, and developmental machinery to extremely different conditions (Boeger and Poulson, 2003; Nielsen and Nielsen, 2006), the epigenetic control system is most likely to provide an effective short-term strategy for amphibious plants to plastically respond to environmental fluctuations by reversibly controlling effector gene expression states depending on environmental conditions.

The genetically identical but phenotypically distinct plants of *A. philoxeroides* provide a suitable model for us to rule out genetic contributions to observed differences, to explore the correlations between environmental stimuli, epigenetic modification and phenotypic variation. Previously, we have investigated the gene expression reaction norms of *A. philoxeroides* in contrasting hydrological conditions, and identified 11 transcriptionally coordinated gene groups associated with environment-induced phenotypic variation in *A. philoxeroides* (Gao et al., 2015). We have also examined genome-wide DNA methylation patterns of *A. philoxeroides* in natural and manipulated habitats, and found that plants of different source populations not only underwent significant morphological changes in common garden environments, but also underwent genome-wide DNA methylation alterations in response to different water treatments, demonstrating a correlation between epigenetic reprogramming and the reversible phenotypic response of *A. philoxeroides* to particular environmental factors (Gao et al., 2010; Deng et al., 2014). In this study, we aim to detect microRNAs potentially involved in the regulation of the plastic responses of *A. philoxeroides* to contrasting hydrological conditions, especially microRNAs associated with enhanced internode elongation growth under submergence. By preliminarily profiling the time-dependent changes of *A. philoxeroides* miRNome in alternate wetting and drying conditions using deep sequencing technology, and validating the most differentially expressed microRNAs by independent quantitative reverse transcription-PCR (qRT-PCR), we identified the submergence-sensitive microRNAs of *A. philoxeroides*, and determined microRNA:mRNA functional pairs and their temporal expression dynamics possibly associated with submergence-induced internode elongation in *A. philoxeroides*. This expanded investigation enhanced our understanding of the role of microRNAs in mediating adaptive developmental response to environmental changes, promoting the study of epigenetic regulation and phenotypic variation in an ecological context.

## Materials and Methods

### Plant Materials, Common Garden Treatments, and Phenotypic Evaluation

Plants of *A. philoxeroides* used in this study were collected from the wild populations in Zhuji, Zhejiang Province (E120°29′ N29°40′) in 2006, and were maintained in the green house of Fudan University, Shanghai (E121°29′ N31°14′). Healthy storage roots from genetically uniform plants were used to produce asexual seedlings. The storage roots were sowed in plastic plates. After the first two new leaves appeared, seedlings of similar sizes were individually transplanted into pots (20 cm in diameter, 15 cm in depth) containing a 1:1:1 mix of peat, vermiculite and sand. After 2 months, the seedlings were subjected to two different types of treatments: pond (submergence) and upland treatments. Two treatments were conducted simultaneously, during the summer months of July and August with about 12 h of daytime, and under identical climatic conditions. In the pond treatment, the potted plants were completely submerged in a series of plastic tanks containing tap water to a depth of 50 cm. Plants were supplied with 1 L water per day in the upland treatment and the soil was kept well-drained (Gao et al., 2015). The length and diameter of the fifth internode from top of 10 individuals were measured under each treatment, at 0, 1, 3, 6, 12, and 120 h from the start of treatments. Tukey’s HSD (honest significant difference) test was performed based on raw data to test differences among sample means for significance. Internode tissues were also harvested and stored in RNALater (Ambion) for RNA extraction. Five replicates from separate plants were collected for each time point and treatment.

### RNA Isolation and Small RNA Sequencing

Total RNAs of each sample were isolated using TRIzol reagent (Invitrogen, Carlsbad, CA, United States), following manufacturer’s instructions. The quality and quantity of obtained RNAs were evaluated on a Bioanalyzer 2100 (Agilent Technologies). RNAs of five replicates for each sample were equivalently mixed according to different time points and treatments. The small RNA libraries were constructed following the standard protocol. In brief, small RNAs with 18–30 nt were separated from the total RNA by polyacrylamide gel electrophoresis. The selected fragments were then ligated to a 5′ RNA adapter and a 3′ RNA adapter, followed by reverse transcription and PCR amplification. PCR products were collected by gel purification and sequenced using the Illumina HiSeq 2000 platform at the Beijing Genomics Institute (BGI), Shenzhen, China. A total of eleven libraries were constructed and sequenced, including an untreated sample library (the control library G0), five upland libraries (D1, D3, D6, D12, and D120) and five pond treatment libraries (W1, W3, W6, W12, and W120). The sequence data were deposited in the United States National Center for Biotechnology Information (NCBI) Sequence Read Archive under accession number SRR5891543 to SRR5891553.

### Identification of Known and Novel microRNAs

Raw reads produced by sequencing were first gone through a data cleaning pipeline to remove low quality reads, contaminating sequences, and reads less than 18 nt. The clean reads were further filtered to remove reads with less than five counts in each library (Lister et al., 2009). Sequences matching non-coding rRNAs, tRNAs, snRNAs, and snoRNAs in the NCBI GenBank^1^ and Rfam^2^ databases were eliminated. The remaining small RNA sequences were aligned against precursor/mature microRNA sequences in miRBase 21^3^ to identify known (conserved) microRNAs in *A. philoxeroides* allowing two mismatches and three gaps. The highest expression microRNA of each mature microRNA family was selected to create a temporary microRNA database. Then clean reads was aligned to this temporary microRNA database and the frequency of each microRNA was generated by summing the count of tags which can align to the temporary microRNA database within two mismatches.

Clean reads that could not be assigned to the aforementioned RNA classes were used to predict potential novel microRNAs in *A. philoxeroides*. Due to the non-availability of the genome sequence of *A. philoxeroides*, the transcriptome of *A. philoxeroides* (Gao et al., 2015) was used to predict novel microRNAs. MIREAP^4^ was used to examine the hairpin structure, the Dicer cleavage site and the minimum free energy of candidate sequences according to the following parameters: (i) minimal microRNA reference sequence length of 20 nt and maximal microRNA reference sequence length of 24 nt; (ii) maximal copy number of microRNAs on the reference was 20; (iii) maximal free energy allowed for a microRNA precursor was -18 kcal/mol; (iv) maximal space between the microRNA and microRNA^∗^ was 300 nt; (v) minimal base pairs of microRNA and microRNA^∗^ was 16; (vi) maximal bulge of microRNA and microRNA^∗^ was 4; (vii) maximal asymmetry of the microRNA/microRNA^∗^ duplex was 4; and (viii) flank sequence length of microRNA precursor was 20.

### Differential Expression Analysis of microRNAs

The frequency of known and novel microRNAs in each library was then normalized to reads per million (RPM) by dividing the read counts of a microRNA by the total number of clean reads of each library, and multiplying this number by 10^5^. The temporal expression pattern of each microRNAs under different treatments was detected by calculating the fold-changes of five upland-treated (D1, D3, D6, D12, and D120) vs. control (G0) and five pond-treated (W1, W3, W6, W12, and W120) samples vs. control (G0), respectively. We considered a fold change of at least 2 (| Log_2_ (treatment/control)| >= 1, *P*-value < 0.05) as an indication of significant change. If the read count of a microRNA was 0 in one sample, its expression value was revised to 0.01; If the RPM of a microRNA was less than 1 in both samples compared, this microRNA was excluded from differentially expression analysis, owing to its low expression (Gao et al., 2012). Pearson’s *r* was used to measure the correlations of microRNA expression in upland- and pond-treated samples, using the R software package for statistical programming, version 3.1.1^6^. To identify differentially expressed microRNAs induced by distinct treatments, the fold-changes of microRNA at different time points of treatment were also calculated between contrast upland- and pond- treated samples. MicroRNAs with the log_2_ fold-change above 1 and a *p*-value < 0.05 (except microRNAs expressed only in upland- treated samples) were defined as submergence-responsive microRNAs.

### Prediction and Functional Annotation of microRNA Targets

To identify the potential targets, all known and novel microRNAs identified in this study were used as queries to search against the *A. philoxeroides* transcriptome (Gao et al., 2015) using the psRNATarget program with default parameter setting (Dai and Zhao, 2011). The BGI microRNA target prediction pipeline which based on the criteria suggested by the two previous reports (Allen et al., 2005; Schwab et al., 2005) was also used for microRNA target prediction. A pool of potential target genes of identified microRNAs was constructed by combining the results of two target prediction tools. By filtering with gene expression profiles (Gao et al., 2015), the target genes that were differentially expressed in contrasting hydrological conditions were identified. GO enrichment analysis was performed using the Bioconductor package topGO^6^. The results of GO enrichment analysis were summarized using WEGO (Ye et al., 2006). The target genes of known and novel submergence-responsive microRNAs were also subjected to GO annotation separately. The web tool REVIGO was used to summarize and visualize the results, which could cluster long, unintelligible lists of GO terms into several representative subsets (Supek et al., 2011). Additionally, not all microRNAs were expressed in all eleven libraries. A part of microRNAs were only expressed in one library. To explore the functional difference of target genes between all-library-expressed microRNAs and one-library-expressed microRNAs, multi-GOEAST analysis was used to compare target genes GO enrichment status between these two categories of microRNAs (Zheng and Wang, 2008).

### qRT-PCR Validation of Transient Co-expression of microRNAs with Their Target Genes

Real-time qPCR was used to validate the co-expression patterns of submergence-responsive microRNAs and their target genes. Four conserved and four novel submergence-responsive microRNAs, were selected and subjected to qRT-PCR analysis. Plant materials used for qRT-PCR validation were collected from another independent common garden experiment. Total RNAs of different samples were extracted using the method mentioned above. For quantification of microRNA expression, the total RNA (1 μg) was first polyadenylated and then reverse transcribed into cDNA with an oligo-dT adapter primer, using the miRcute microRNA First-Strand cDNA Synthesis Kit (TIANGEN, Beijing, China). qRT-PCR was carried out using the miRcute microRNA qPCR Detection kit (SYBR Green) (TIANGEN, Beijing, China) following manufacturer’s instructions. The specific forward primer designed for each microRNA and the universal reverse primer provided by kit were used for amplification. For quantification of target mRNAs by qRT-PCR, the total RNA of each sample was reverse transcribed into cDNA using PrimeScript^TM^ RT Master Mix (Perfect Real Time) (Takara, Dalian, China). qRT-PCR was performed with LightCycler^®^ 480 SYBR Green I Master (Roche, United States) following manufacturer’s instructions. Ubiquitin C (UBC) was used as an internal control. The primers used for qRT-PCR were listed in Supplementary Table S1. All reactions were performed in triplicate for each sample on LightCycler^®^ 96 System. The relative time-course changes in microRNA and mRNA expression levels between pond and upland treatments were quantified using the 2^-ΔΔC_t_^ method (Livak and Schmittgen, 2001).

### Target Validation by 5′ RLM-RACE

The 5′ RLM-RACE (RNA ligase-mediated 5′ rapid amplification of cDNA ends) method was used for target validation (Park and Shin, 2014). The total RNAs of pond-treated samples were isolated using TRIzol reagent (Invitrogen, Carlsbad, CA, United States) following manufacturer’s instructions. mRNAs were separated from the total RNA using PolyA Tract mRNA Isolation System III (Promega, United States), and were then used to perform 5′ RLM-RACE using GeneRacer^TM^ Kit (Invitrogen, Carlsbad, CA, United States), according to the instructions of the manufacturer. Gene-specific nested reverse primers (listed in Supplementary Table S1) were used in combination with the 5′ primer provided by the kit to amplify the cleaved transcripts. Amplicons were purified using the AxyPrep DNA gel extraction kit (Axygen, United States), cloned into the pEASY-T3 vector (Transgene, Beijing, China), and sequenced.

## Results

### Phenotypical Variation of *A. philoxeroides* under Pond and Upland Treatments

**FIGURE 1:**
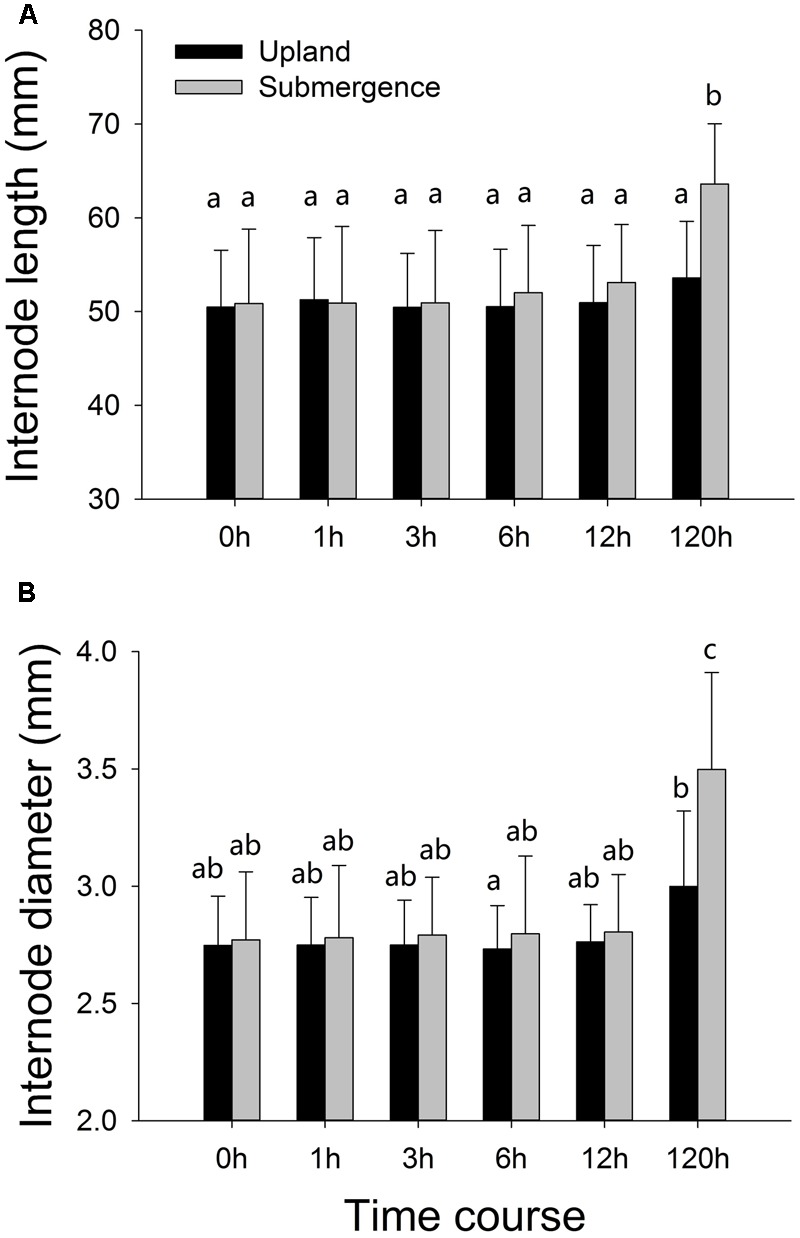
Phenotypic variation of *Alternanthera philoxeroides* in response to upland and pond treatments. **(A)** Internode length; **(B)** internode diameter. Quoted values are means ± SE (*N* = 10). Different letters indicate significant differences among groups.

Internode elongation and increases in stem diameter were not significant between plants under distinct treatments at the first four time points (1, 3, 6, and 12 h). One hundred and twenty hours later, significant differences were found between plants subjected to pond and upland treatments, respectively, with the plants growing in pond environment exhibiting significantly enhanced internode elongation, accompanied by increases in stem diameter (**Figure 1**).

### Conserved and Novel microRNAs of *A. philoxeroides*

Illumina HiSeq was used to sequence the small RNA libraries. On average, 12.98 million (11719363 to 14236593) reads were obtained for each library. After stringent quality check and data cleaning, approximately 12.73 million (11596962 to 14093418) clean reads were left for each library (Supplementary Table S2). Clean reads were classified as different types of non-coding transcripts, such as rRNA, tRNA, snRNA, snoRNA, microRNA, and unannotated reads. The numbers of each class in each library were shown in Supplementary Table S3. The 24 nt class was the most abundant group of sRNAs in all libraries followed by 21 nt sequences (**Supplementary Figure S1**). Conserved microRNAs were identified by BLAST searching against the miRBase database. A total of 220 known microRNAs belonging to 76 families were identified. The number of members varied in different microRNA families. The MIR166 family had the most abundant members in *A. philoxeroides* (nine members), followed by MIR159 (six members). Four families (MIR167_1, MIR169_1, MIR172, and MIR396) contained five members. Fifty-two families (68.4%) had only one representative in *A. philoxeroides*. Investigation of the distribution of the 220 known microRNAs among different libraries revealed that 113 (51.4%) microRNAs were expressed not only in the pond- and upland-treated samples, but also in the control library G0. Among them, 66 microRNAs were constantly expressed in all libraries across different time points. These microRNAs came from 32 families, and about a half of these families were well-conserved across the plant kingdom, such as MIR159 (three members) and MIR166 (six members). Some known microRNAs were found to be specific to the control (3), the pond-treated (37) and the upland-treated (18) samples, respectively (**Supplementary Figure S2A**). The microRNAs specifically expressed in the pond-treated samples mostly belonged to the less-conserved microRNA families such as MIR824 (one member), MIR837 (one member) and MIR7982 (1 member), yet some of them came from well-conserved families such as MIR169_1 (miR169d-5p, miR169n-3p, and miR169i).

The MIREAP prediction software was used to identify potentially novel microRNAs in *A. philoxeroides*. A total of 81 novel microRNAs were identified. The length of the novel microRNA precursors varied from 72 to 345 nt, with an average of 131 nt. The average minimum free energy (MFE) ranged from -107.9 to -19 kcal/mol, with an average of -37.93 kcal/mol. The sequences and hairpin secondary structures of novel microRNAs were shown in Supplementary Table S4. Among 81 newly identified microRNAs, 12 (14.8%) were expressed in both the control and the pond- and upland-treated samples. 16 (19.75%) were shared by the pond- and upland-treated samples. 5 (6.17%), 21 (25.92%), and 24 (29.62%) were specific to the control, pond- and upland-treated samples, respectively (**Supplementary Figure S2B**).

### Expression Dynamics of Identified microRNAs

**FIGURE 2:**
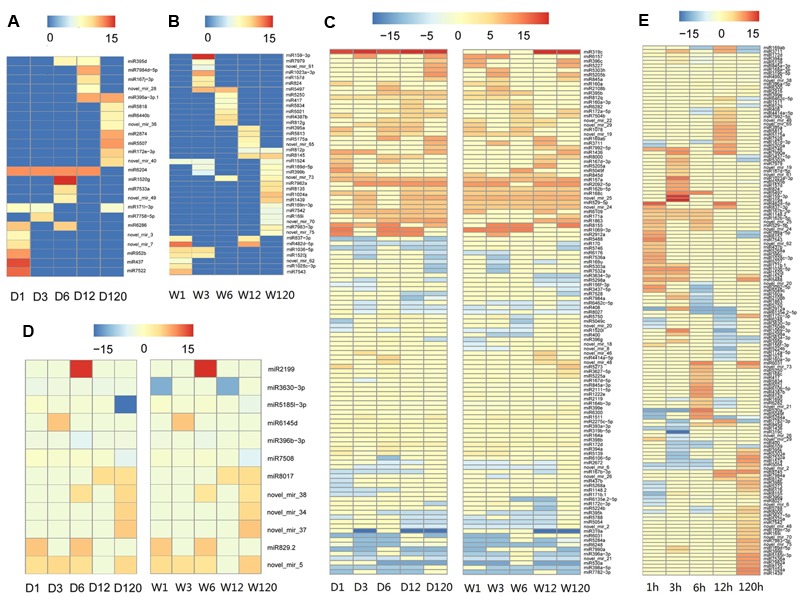
Temporal patterns of microRNA expression in *A. philoxeroides* in contrasting hydrological habitats. **(A)** microRNAs specifically expressed in the upland-treated samples; **(B)** microRNAs specifically expressed in the pond-treated samples; **(C)** microRNAs expressed in both pond- and upland-treated samples but showing distinct expression dynamics; **(D)** microRNAs showing consistent expression patterns between the upland- and pond-treated samples; **(E)** microRNAs differentially expressed between the upland- and pond-treated samples. The color bars show log2 fold change value between the upland-/pond-treated samples and G0 sample **(A–D)** or between contrast pond- and upland-treated samples **(E)**. The data is presented in Supplementary Tables S5, S6.

Time course expression profiles revealed that, among 301 known and novel microRNAs identified in *A. philoxeroides*, 70 (66 known and 4 novel) microRNAs were constantly expressed in all libraries across different time points of the treatments. The rest of microRNAs showed varied expression between different libraries. Ninety-four (47 known and 47 novel) microRNAs were expressed only in a single library. Comparison of the expression profiles led to the identification of 179 (153 known and 26 novel) microRNAs that were differentially expressed between the pond- and upland-treated samples. Of them, 24 and 40 microRNAs were specifically expressed in the upland- (**Figure 2A** and Supplementary Table S5) and pond-treated (**Figure 2B** and Supplementary Table S5) samples, respectively. The rest 115 microRNAs were expressed in both pond- and upland-treated samples but with different expression patterns (**Figure 2C** and Supplementary Table S5). Twelve microRNAs showed consistent expression patterns between the upland- and pond-treated samples (*r* > 0.9, *p* < 0.05, **Figure 2D** and Supplementary Table S5). To identify submergence-responsive microRNAs, the upland-treated samples were used as controls to calculate the relative expression value of microRNAs between treatments. A total of 146 (128 known and 18 novel) microRNAs were identified as submergence-responsive microRNAs (Supplementary Table S6), and their expression levels were significantly altered at different time points of the treatments (**Figure 2E**). Eighty-four microRNAs (e.g., miR1023a-3p, miR3630-3p, and novel_mir_25) responded to the pond treatment at the early stage of the treatment (1–12 h), and 19 microRNAs (e.g., miR1439, miR7542 and novel_mir_48) responded at the later stage (120 h). Forty-three microRNAs (e.g., miR167b-3p, miR5488, and novel_mir_73) exhibited varied patterns of differential expression between the upland- and pond-treated samples at different stages of the treatments.

### Predicted Targets of Known and Novel microRNAs

**FIGURE 3:**
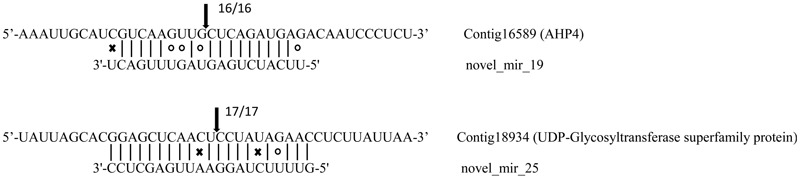
Targets of microRNAs verified by 5′ RLM-RACE. Arrows indicate the cleavage sites with the frequency of clones shown beside. Vertical dash lines represent the Watson-Crick pairing. Circles and crosses indicate the G:U wobble pairing and mismatched base pairing, respectively.

Based on *in silico* target prediction, a total of 2503 genes were identified as the potential target genes of the known and novel *A. philoxeroides* microRNAs, in which 1439 were found to be differentially expressed in pond and upland environments. Among these differentially expressed genes, 732 were predicted as the targets of known submergence-responsive microRNAs, and 132 were targeted by novel submergence-responsive microRNAs. The cleavage sites of two target genes (Contig16589_AHP4 and Contig18934_UDP-Glycosyltransferase superfamily protein) recognized by novel_mir_19 and novel_mir_25, respectively, were confirmed by 5′ RLM-RACE experiments. The cleavage site was concentrated at the 10th nucleotide from the 5′ end of each microRNA (**Figure 3**).

The results of the GO term enrichment analysis showed that the microRNAs ubiquitously expressed in all samples were more likely to regulate genes involved in basic life processes, whereas the targets of transiently expressed microRNAs were much more versatile than those of constantly expressed microRNAs (**Supplementary Figure S3**). The target genes regulated by submergence-responsive microRNAs were involved in a variety of cellular and physiological processes, such as anatomical structure formation, response to stimulus and developmental or metabolic process, and the distributions of the different GO categories are shown in **Supplementary Figure S4**. Interestingly, genes targeted by known microRNAs seem more likely to be engaged in cell wall metabolism and remodeling in response to flooding stress, including genes participating in cell wall organization, lignin catabolism, and UDP-D-xylose biosynthesis (**Supplementary Figure S5A**). Instead, targets of novel microRNAs mainly participated in plant hormone response and epigenetic processes, such as cellular response to gibberellin and regulation of DNA methylation (**Supplementary Figure S5B**).

### Co-expression Patterns between microRNAs and Their Target mRNAs

**FIGURE 4:**
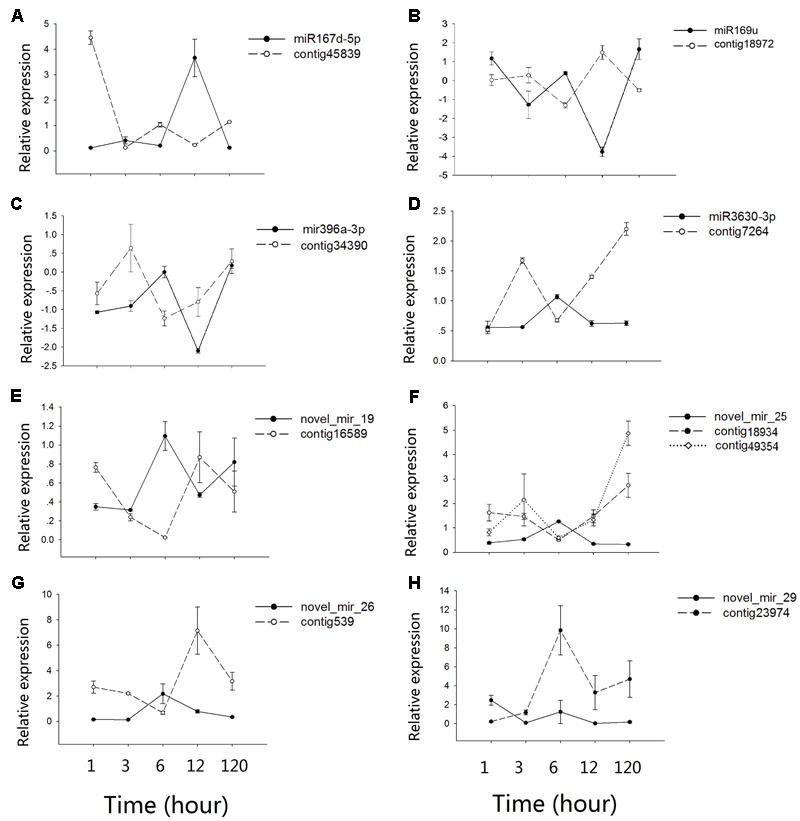
Co-expression patterns between microRNAs and their targets. **(A–H)** shows the qRT-PCR results for different microRNAs-mRNAs pairs. Values along the *y*-axis represent the differential expression log_2_ ratios between pond and upland samples. Relative expression quantification of microRNAs and their target genes was carried out using the 2^-Δ ΔC_t_^ method, with UBC gene as internal control. Error bars represent mean ± SD (*n* = 3).

Contrasting hydrologic treatments led to global changes in microRNA and mRNA expression patterns in *A. philoxeroides*. By integrating expression profiles of microRNA and mRNA in conjunction with the predicted microRNA targets, we identified eight microRNA:mRNA pairs potentially involved in the regulation of internode elongation under submergence. Quantitative analysis using qRT-PCR confirmed the inverse expression patterns between microRNAs and their target mRNAs, with the exception of novel_mir_29:Contig23974 (**Figure 4**). Mir167d-5p was dramatically induced after 12 h of submergence following a subtle up-regulation at 3 h of the pond treatment, and then down-regulated. Its target mRNA cotig45839 showed the expected negative regulation pattern (**Figure 4A**). mir3630-3p, novel_mir_25 and novel_mir_26 were transiently up-regulated at 6 h of submergence and returned to normal levels at 12 h of the pond treatment, with the expression levels of their target mRNAs (contig7264, contig18934/contig49354, and contig539) being negatively correlated with those of the corresponding microRNAs (**Figures 4D,F,G**). miR169u, miR396a-3p and novel_mir_19 exhibited fluctuate expression patterns. They were up- and down-regulated at the early stage (1–12 h) of the treatment, and then up again during the late phase (120 h) (**Figures 4B,C,E**). The co-expression pattern of novel_mir_29 with its target gene Contig23974 was distinct from others. Although the expression of novel_mir_29 was differentially regulated at different time points of the treatment, the expression level of the putative target transcript was not changed accordingly in an inversely correlated pattern (**Figure 4H**).

## Discussion

Due to their sessile lifestyle, plants have developed a remarkable plasticity for shaping their body plan to adapt to changes in their immediate environment. The high sensitivity of microRNAs to environmental perturbations and their significant role in readjusting the expression levels of genes and phenotypic profiles under changing environments have made them the best candidates as dynamic regulators of phenotypic plasticity (Rubio-Somoza and Weigel, 2011).

Genome-wide perspective of miRNAome in response to pond and upland treatments revealed 146 submergence-responsive microRNAs belonging to 49 microRNA families in *A. philoxeroides*. The members from 19 families (such as MIR159, MIR167_1, MIR169, MIR396, MIR482, MIR530, and MIR3630) were not only involved in responses to water-logging/flooding in *A. philoxeroides*, but also in other plant species. For instance, gene expression profiling and qRT-PCR analyses revealed the varied expression patterns of miR169u and miR396a-3p in *A. philoxeroides* during submergence. The similar patterns were also found in *Nelumbo nucifera* (Jin et al., 2017) and *Zea mays* (Zhang et al., 2008; Liu et al., 2012). Three members of the miR167_1 family (miR167b-3p, miR167d-5p, and miR167d-3p) showed significantly altered expression between the pond- and upland-treated samples in *A. philoxeroides*. Similarly, the members from this family were differentially expressed under flooding condition in *P. tomentosa* (Ren et al., 2012). These results suggest the common roles of these microRNAs in different plant species. The submergence-responsive microRNAs identified in *A. philoxeroides* can also be induced by components of submergence, such as hypoxic stress (e.g., miR159, miR169, miR319, miR394, and miR395) (Licausi et al., 2011), elevated CO_2_ concentration (e.g., miR156, miR157, miR172, and miR160) (May et al., 2013), darkness (e.g., miR160, miR167, miR171, miR396, and miR398) (Huo et al., 2015) and ethylene entrapment (e.g., miR399, miR408, miR482, miR4414, and miR5139) (Pei et al., 2013).

Target prediction and functional annotation of the submergence-responsive microRNAs recognized microRNAs potentially associated with plastic internode elongation in *A. philoxeroides* upon submergence. The target of novel_mir_26 was homeobox protein 22 (HB22), which has been shown to be associated with the synthesis of gibberellic acid (GA) in *Arabidopsis* (Bueso et al., 2014). The primary action of GA in stem growth was on cell elongation (Arney and Mancinelli, 1966). The expression of *A. philoxeroides* HB22 (Contig539) was significantly up-regulated at 12 h of the pond treatment accompanied by down-regulation of novel_mir_26, that might promote internode elongation in submerged plants by enhancing GA biosynthesis (Voesenek and Bailey-Serres, 2015). Both cytokinin and brassinosteroid (BR) were also implicated in stem elongation growth (Azpiroz et al., 1998; Hansen et al., 2009). The target of novel_mir_19 in *A. philoxeroides* was contig16589, which was homologous to *Arabidopsis* AHP4 and potentially involved in cytokinin signaling pathway (Hutchison et al., 2006). Novel_mir_19 and its target exhibited a significant inverse expression pattern under the pond treatment (**Figure 4E**), and the target cleavage site was verified by 5′ RLM-RACE. *A. philoxeroides* contig2589, a homolog of *Arabidopsis* CDL1 that positively regulates BR signaling (Kim et al., 2011) and stem elongation (Peeters et al., 2002), was predicted as the target of miR398a-5p. The expression levels of contig2589 and miR398a-5p were also changed accordingly in an inversely correlated pattern according to the expression profile.

Abscisic acid (ABA) antagonizes the effects of GA and other phytohormones in numerous processes during plant growth and development (Bailey-Serres and Voesenek, 2010; Finkelstein, 2013). Growth-regulating factors (GRFs), a conserved class of plant-specific transcription factors, participate in ABA signaling pathway as a transcriptional repressor of ABA- and osmotic-stress responsive genes (Kim et al., 2012). The expression of several GRFs is controlled by miR396 (Omidbakhshfard et al., 2015), functioning as a regulator of hormone homeostasis and signaling under flooding stress (Zhang et al., 2008). In our study, miR396-3p presented fluctuate expression patterns opposite to contig34390 (GRF6) during the pond treatment (**Figure 4C**), indicating the interplay between ABA and other phytohormones in *A. philoxeroides* upon submergence. Previous studies have shown that interactions between different plant hormones play a central role in generating plastic phenotypic responses to environmental variation (Sultan, 2000; Lema, 2014), including in promoting shoot elongation upon submergence (Van Der Straeten et al., 2001; Voesenek et al., 2003; Cox et al., 2004, 2006; Jackson, 2008; Van Veen et al., 2013). The detection of dynamic expressions of microRNAs and their corresponding targets associated with different hormone signaling pathways in this study possibly suggests the potential role of *A. philoxeroides* microRNAs in coordinating multiple phytohormone responses during the submergence treatment. The crosstalk between microRNAs and hormone signaling cascades deserves better attention while studying submergence-induced elongation growth in flooding-tolerant plants.

Previous gene expression profiling showed that a large number of genes associated with different processes of cell wall strengthening, such as the xylan biosynthetic process, the lignin biosynthetic process, secondary cell wall biogenesis, etc., exhibited a significant “up-down” pattern in the early stage of the pond treatment, suggesting a “Just-in-time” cell wall thickening in response to submergence though its function remaining unclear (Gao et al., 2015). In coincidence with the transient cell wall strengthening, we found in this study that NF-YA3 (*A. philoxeroides* contig18972), a transcript factor which has been shown to impair cell elongation (Leyva-González et al., 2012), was transiently up-regulated in the early stage of the pond treatment (**Figure 4B**), inversely correlated with the expression of the corresponding miR169u. In contrast, the expression of SKS5 (*A. philoxeroides* contig45839), a gene that has been identified as a cell wall-related gene (Mutwil et al., 2009) and was expressed most strongly in expanding tissues (Sedbrook et al., 2002), was highly repressed in the early stage of the pond treatment accompanied by induced and transient up-regulation of the corresponding miR167d-5p (**Figure 4A**). The transient down-regulation of the cell wall expansion-promoting gene SKS5 was consistent with the transient up-regulation of cell wall strengthening genes. microRNAs, while functioning as a trigger of hormone response to external environment, may also coordinate the expression of genes associated with different processes of cell wall modification under submergence.

However, the relationships between microRNAs and their target genes was not always straightforward. One microRNA seemed to be able to regulate more than one target gene and, in turn, one gene might be regulated by more than one microRNA. For example, both contig49354 (DUF579) and contig18934 (UDP-Glycosyltransferase superfamily protein) were identified as the targets of novel_mir_25 and showed similar inverse relationships with novel_mir_25 in expression (**Figure 4F**), while the expression of contig2773 (SPL3) was regulated by miR157a and miR4387b, respectively, at different time points of the treatment. We also found a positive relation between the expression of novel_mir_29 and its target Contig23974 (vacuolar invertase, VI2), a gene that has long been considered as a major player in cell expansion (Wang et al., 2010) (**Figure 4H**). The most striking feature revealed by time course expression profiling was the fluctuating pattern in microRNA expression. Most of the differentially expressed microRNAs were transiently up-/down-regulated at certain time points during the treatments. More than half of the submergence-responsive microRNAs (e.g., miR1023a-3p, miR3630-3p, and novel_mir_25) responded to the pond treatment at the early stage of the treatment (1–12 h). About one-third of the microRNAs were differentially expressed at the 1st hour from the start of treatments, while 19 microRNAs (e.g., miR1439, miR7542, and novel_mir_48) responded to the treatment 120 h later. The fine-scale temporal changes in microRNA expression highlight the importance of time-series sampling in identifying stress-responsive microRNAs and analyzing their role in stress response/tolerance. Incomplete sampling might give a biased conclusion about the microRNA’s role in readjusting the expression levels of genes and phenotypic profiles under changing environments.

## Author Contributions

GL performed the wet lab work and data analysis, and drafted the manuscript. YD, YG, and CZ helped to set up experiments. YW, WZ, and ZS participated in data analysis. LG and JY conceived the idea, participated in the design of the study and finalized the manuscript. All of the authors read and approved the final manuscript.

## Conflict of Interest Statement

The authors declare that the research was conducted in the absence of any commercial or financial relationships that could be construed as a potential conflict of interest. The reviewer OV-L and handling Editor declared their shared affiliation.

## Footnotes

1 https://www.ncbi.nlm.nih.gov/genbank/

2 http://www.sanger.ac.uk/Software/Rfam

3 http://www.mirbase.org/

4 https://sourceforge.net/projects/mireap/

5 http://bioconductor.org/packages/release/bioc/html/topGO.html

6 http://www.r-project.org/

## Supplementary Material

FIGURE S1 Length distribution of small RNAs in each library.

FIGURE S2 microRNAs expressed in different sample libraries. **(A)** Known microRNAs; **(B)** novel microRNAs.

FIGURE S3 Results of multi-GOEAST analysis, showing that targets of ubiquitously expressed microRNAs were more likely to be involved in basic life processes (red), whereas the targets of transiently expressed microRNAs were much more versatile (green). Yellow boxes represent common GO terms enriched in both categories of targets. The degree of color saturation indicates the significance of enrichment for corresponding GO terms. Arrows represent connections between different GO terms. Red arrows represent direct relationships between two GO terms, and black arrows (solid and dashed) represent indirect relationships between GO terms.

FIGURE S4 Gene Ontology classifications of the target genes based on cellular component, molecular function, and biological process.

FIGURE S5 Summarized biological process of known **(A)** and novel **(B)** microRNA target genes. Loosely related terms were jointed into “super-clusters” by different colors with a representative showed in middle. Size of the rectangles was adjusted to *p*-value of GO annotation.
