# Supplementary figures and images for: Differentially Expressed microRNAs and Target Genes Associated with Plastic Internode Elongation in Alternanthera philoxeroides in Contrasting Hydrological Habitats
Source: Front Plant Sci. 2017 Dec 5;8:2078. doi: 10.3389/fpls.2017.02078 (PMC5723390; doi:10.3389/fpls.2017.02078)

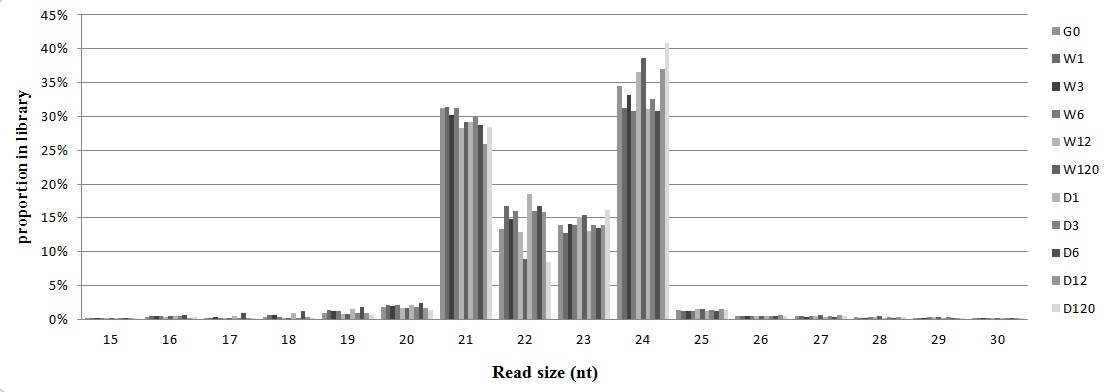

Supplement: FIGURE S1 — Length distribution of small RNAs in each library. [file Image_1.JPEG]

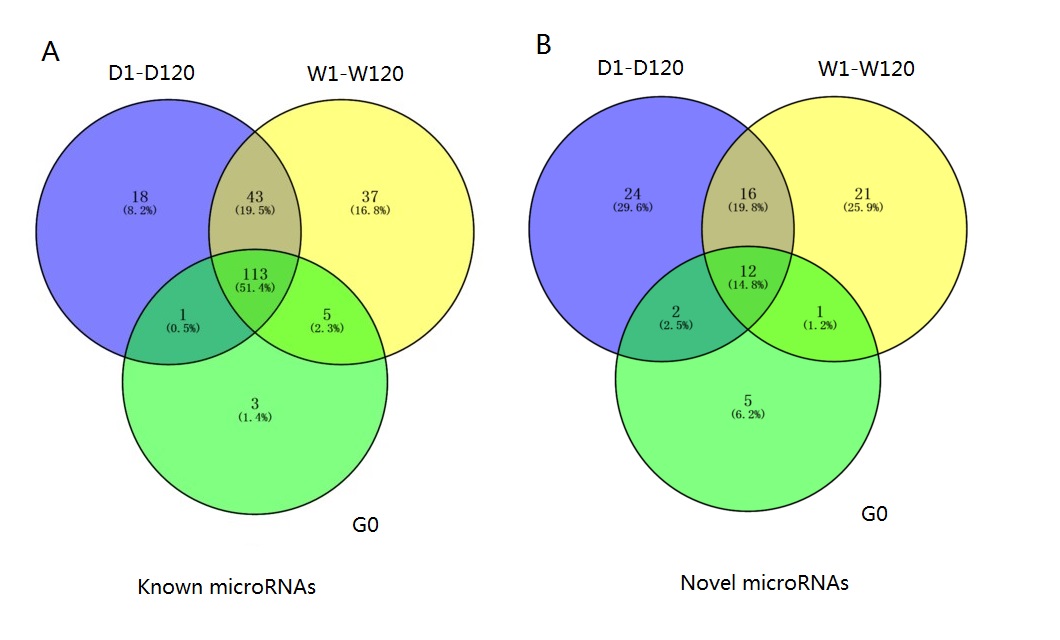

Supplement: FIGURE S2 — microRNAs expressed in different sample libraries. (A) Known microRNAs; (B) novel microRNAs. [file Image_2.JPEG]

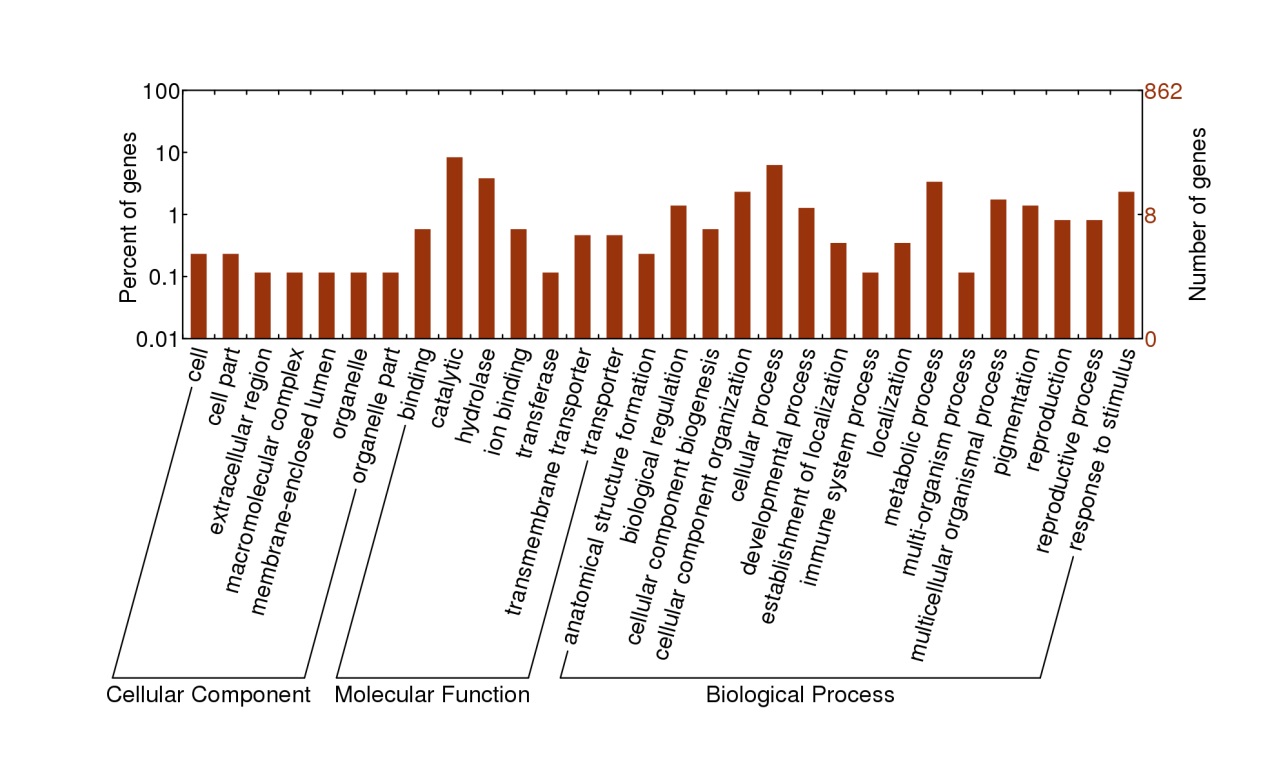

Supplement: FIGURE S4 — Gene Ontology classifications of the target genes based on cellular component, molecular function, and biological process. [file Image_4.JPEG]

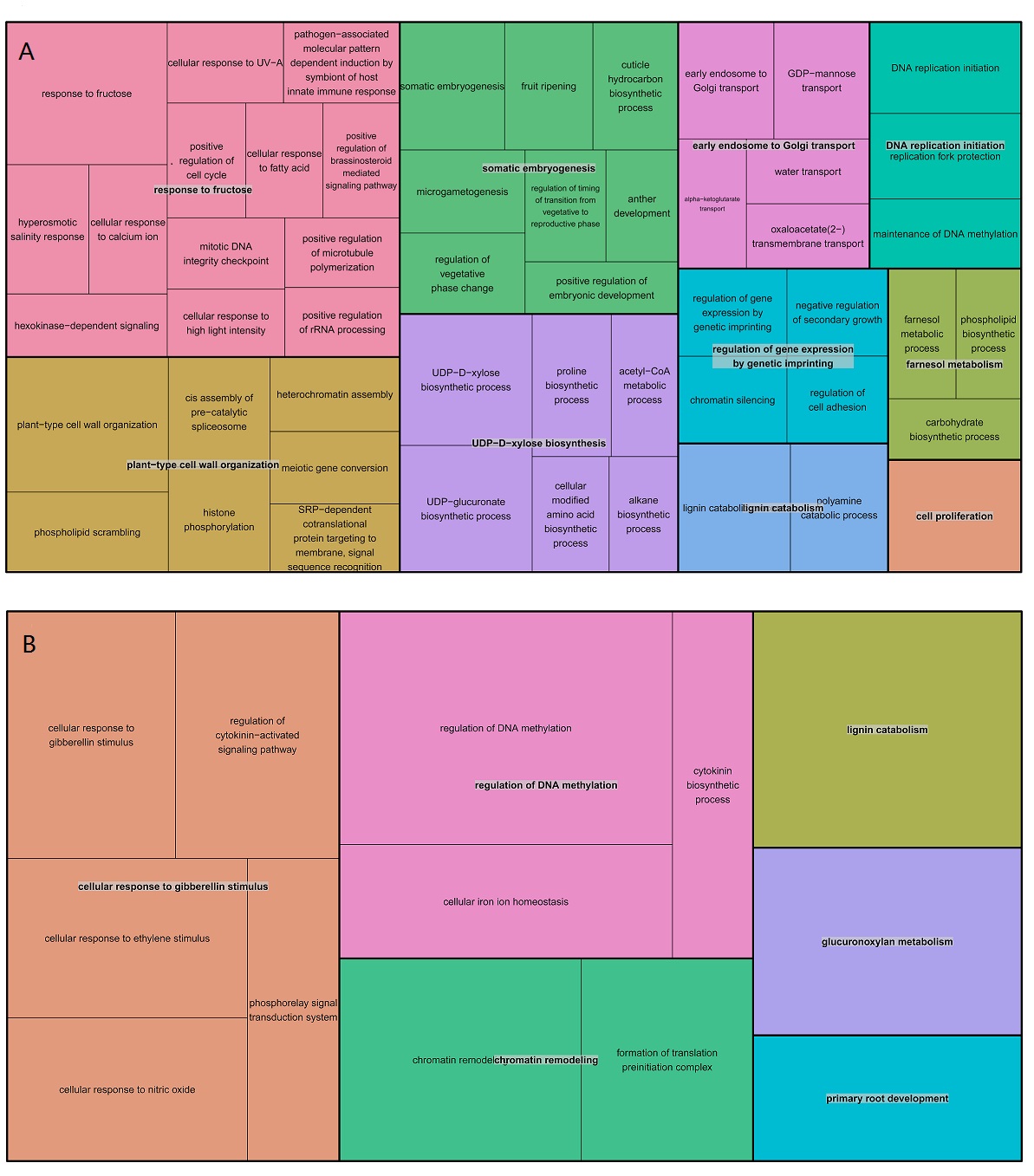

Supplement: FIGURE S5 — Summarized biological process of known (A) and novel (B) microRNA target genes. Loosely related terms were jointed into “super-clusters” by different colors with a representative showed in middle. Size of the rectangles was adjusted to p-value of GO annotation. [file Image_5.JPEG]
